# Chinese herbal medicine Tangshen Formula treatment of patients with type 2 diabetic kidney disease with macroalbuminuria: study protocol for a randomized controlled trial

**DOI:** 10.1186/s13063-016-1385-2

**Published:** 2016-05-23

**Authors:** Meihua Yan, Yumin Wen, Liping Yang, Xi’ai Wu, Xiaoguang Lu, Bingxuan Zhang, Weiping Huang, Ping Li

**Affiliations:** Beijing Key Lab for Immune-Mediated Inflammatory Diseases, Institute of Clinical Medical Science, China-Japan Friendship Hospital, 2 East Yinghua Road, Chaoyang District Beijing, 100029 China; Department of Nephrology, Guang’anmen Hospital of China Academy of Traditional Chinese Medical Sciences, 5 Beixiange, Xicheng District Beijing, 100053 China; Beijing Qihuang Drug Clinical Research Center, 9 Guang’an Road, Fengtai District Beijing, 100055 China

**Keywords:** Chinese herbal medicine, Diabetic kidney disease, Macroalbuminuria, Randomized controlled trial, Tangshen Formula

## Abstract

**Background:**

Diabetic kidney disease (DKD) is one of the most common microvascular complications of diabetes mellitus and the main cause of end-stage renal disease. Present medications for DKD are not entirely satisfactory. Preliminary studies indicate that the Chinese herbal formula Tangshen Formula (TSF) appears to decrease the proteinuria and improve the estimated glomerular filtration rate (eGFR) in DKD patients.

**Methods/design:**

This trial is a five-center, randomized, double-blind, placebo-controlled study. DKD patients with a 24-h urinary protein (24 h UP) level between 0.5 and 3.5 g and serum creatinine < 265 μmol/L (3 mg/dl) will be included. A sample size of 144 participants will be randomly distributed into the treatment group (TSF plus irbesartan) and the control group (placebo plus irbesartan) at a ratio of 1:1. The study duration will be 50 weeks, comprising a 2-week run-in period, 24 weeks of intervention, and 24 weeks of follow-up. The primary endpoint will be the 24 h UP. Secondary endpoints will be an evaluation of renal function, management of blood lipids, improvement in traditional Chinese medicine symptoms, and safety assessments. Adverse events will also be evaluated.

**Discussion:**

This study will provide evidence for the effectiveness and safety of TSF compared to placebo in treating DKD patients with macroalbuminuria.

**Trial registration:**

Chinese Clinical Trials Registry, ChiCTR-TRC-13003566. Registered 9 August 2013.

**Electronic supplementary material:**

The online version of this article (doi:10.1186/s13063-016-1385-2) contains supplementary material, which is available to authorized users.

## Background

Diabetic kidney disease (DKD) is a major microvascular complication of both type 1 and type 2 diabetes mellitus (DM) [1]. However, in type 1 diabetics, DKD typically does not present until the second decade after diagnosis of diabetes, whereas in some type 2 diabetics, DKD is already evident at the time of diagnosis of diabetes because of other chronic instigators of kidney injury such as aging, hypertension, and abnormal lipid levels [2, 3]. In China, DKD is the second leading cause of end-stage renal disease (ESRD) after glomerulonephropathy [4] and is a leading cause of dialysis in developed countries such as Japan and the USA [5, 6]. The economic burden of DKD is substantial [7, 8]. Angiotensin-converting enzyme inhibitors (ACEIs) and angiotensin receptor blockers (ARBs) have been the agents of choice in slowing the progression of DKD. However, as seemingly effective as they are, these drugs have side effects such as rhinitis, persistent cough, and angioedema, which cause patients to discontinue therapy in some cases [9, 10]. Thus, it is clear that an unmet need exists for developing renoprotective therapies that are not only effective but also well tolerated.

Recent studies appear to show that certain Chinese herbs have renoprotective effects against chronic kidney disease (CKD) [11] and DM [12]. Hence, to assess the efficacy and safety of these herbs in DKD treatment, a well-designed, randomized, controlled trial (RCT) is needed.

Traditional Chinese medicine (TCM) is a medical system founded on syndrome pattern differentiation. Clarifying the main TCM syndrome type of DKD is essential for its treatment. We employed the Delphi method to investigate the most common signs and symptoms of DKD as propounded by TCM nephrology experts. From three rounds of questionnaires, we concluded that deficiency of both *qi* and *yin* with blood stasis is the main TCM syndrome type of DKD [13]. This result was in accordance with findings of other studies [14, 15] and was verified in our subsequent cross-sectional study of 350 DKD patients [16]. Based on this main syndrome type and empirical evidence from Chinese medicine practitioners, the herbs in Tangshen Formula (TSF) were chosen to treat DKD. The TCM function of the herbs in TSF is to replenish *qi* and *yin* and to promote blood circulation. Ensuing studies suggested that TSF treatment could decrease the urinary albumin excretion rate (UAER) and reduce glomerulosclerotic and interstitial fibrosis indices in both Otsuka Long-Evans Tokushima fatty (OLETF) spontaneously diabetic rats [17] and streptozotocin combined with uninephrectomy-induced type 1 diabetic rats [18]. Further research indicated that in DKD rats, the therapeutic effects of TSF might at least be partially due to its anti-inflammatory action of downregulating tumor necrosis factor α and upregulating pro-inflammatory cytokine interleukin-10 [19], as well as its antifibrotic action of inhibiting expression of transforming growth factor beta 1 (TGF-β1) in renal tissue, enhancing the expression of matrix metallopeptidase 9 (MMP-9), and reducing the expression of collagen type IV [18]. Another study of ours also found that TSF appears to exert a renal protective effect by improving lipid metabolism [20]. Our preliminary RCT indicated that TSF treatment might improve eGFR and decrease proteinuria, especially in patients with macroalbuminuria [21].

The current study is designed to validate whether or not TSF is safe and efficacious in the treatment of DKD patients with macroalbuminuria.

## Methods/design

### Ethics, consent, and permissions

The protocol (version identifier: TSF-3.0 (20130512)) has been approved by the ethics committee of the China-Japan Friendship Hospital (Approval No. 2013–46) and is registered with the Chinese Clinical Trial Registry (http://www.chictr.org.cn) under registration number ChiCTR-TRC-13003566. The study will be conducted in accordance with the principles of good clinical practice, i.e., the Declaration of Helsinki (2004 version). The trial will be reported according to the Standard Protocol Items: Recommendations for Intervention Trials (SPIRIT) (see Additional file 1). All patients will be informed about the purpose of the trial, the risks, and the benefits, and informed consent (see Additional file 2) will be obtained from all participants prior to entry into the trial. All visits will be documented in a case report form (CRF).

### Study design and setting

**Fig. 1:**
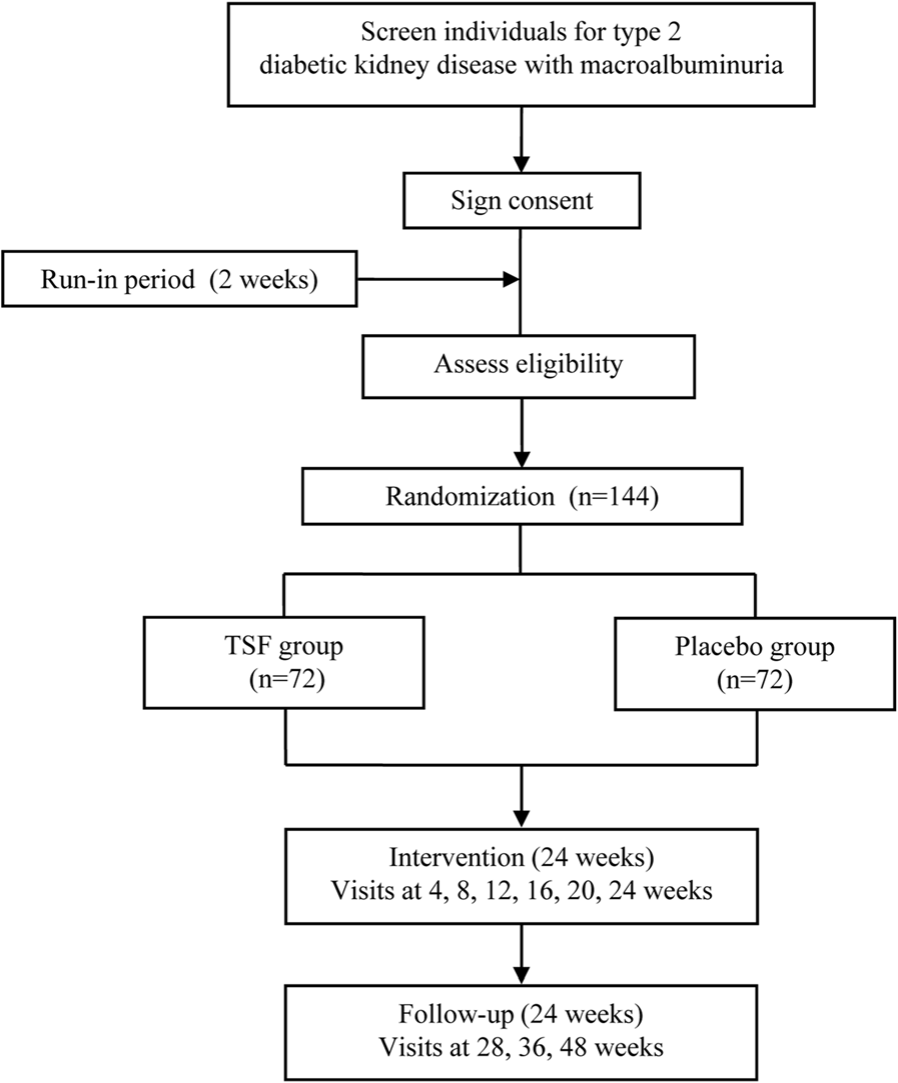
Flow diagram of progress through the study

This trial will be a randomized, placebo-controlled, multicenter trial with two parallel groups. Investigators, the statistician, and participants will be blinded. The trial flow is illustrated in Fig. 1. Five hospitals in China will participate in the study: the China-Japan Friendship Hospital, Beijing; the First Teaching Hospital of Tianjin University of Traditional Chinese Medicine, Tianjin; the Hangzhou Hospital of Traditional Chinese Medicine Affiliated to Zhejiang University of Chinese Medicine, Hangzhou; the Shaanxi Provincial Hospital of Traditional Chinese Medicine, Xi’an; and the Longhua Hospital Affiliated to Shanghai University of Traditional Chinese Medicine, Shanghai.

### Eligibility criteria

#### Diagnostic criteria

The diagnosis of type 2 diabetes will be based on the American Diabetes Association (ADA) criteria [22], and DKD will be defined by the National Kidney Foundation Kidney Disease Outcomes Quality Initiative (NKF K-DOQI) guidelines [23].

#### TCM syndrome pattern differentiation

The Chinese syndrome pattern differentiation type of deficiency of both *qi* and *yin* with blood stasis will be based on guidelines delineated in *Clinical Research of New Investigational Drugs in Traditional Chinese Medicine* [24]. The diagnostic standards are as follows:

1. Primary signs and symptoms include fatigue, listlessness, weakness, and soreness of the low back and knees; heat sensation in the palms and soles; and dry mouth and throat.
2. Secondary signs and symptoms include catching cold easily, pale complexion, irritability, numbness, edema, frequent urination at night, constipation, and hematuria.

Participants will be diagnosed with deficiency of both *qi* and *yin* with blood stasis syndrome if they have two or more of the primary signs/symptoms and at least two of the secondary signs/symptoms. Investigators at each center will administer a symptom assessment survey to every participant (Table 1). This will ensure standardized scoring across centers.

**Table 1:** Traditional Chinese medicine (TCM) symptom assessment survey

Fatigue	□ 0: None □ 2: Cannot sustain heavy work □ 4: Can manage mild intensity work □ 6: Can only do daily activities
Weakness and soreness of the low back and knees	□ 0: None □ 2: Occasional □ 4: Need to alter body position for relief □ 6: Persistent pain, need to take pain medication
Heat sensation in the palms and soles	□ 0: None □ 2: Occasional □ 4: Desire to expose extremities □ 6: Desire to hold something cold
Dry mouth and throat	□ 0: None □ 2: Mild □ 4: Decreased saliva production □ 6: Severe thirst with constant need to drink fluids
Qi deficiency and listlessness	□ 0: None □ 2: Shortness of breath after mild activity □ 4: Shortness of breath after moderate activity □ 6: Unable to talk or catch breath even without activity
Catch cold easily	□ 0: No colds □ 1: At least 6 times per year □ 2: At least 10 times per year □ 3: 12 or more times per year
Pale complexion	□ 0: None □ 1: Mild □ 2: Moderate □ 3: Pale or dark yellow complexion
Irritability	□ 0: None □ 1: Occasional □ 2: Easily irritated, but able to gain self-control □ 3: Severe agitation, unable to gain self-control
Numbness	□ 0: None □ 1: Hands and feet □ 2: Limbs □ 3: Entire body
Edema	□ 0: None □ 1: Palpebral edema in the morning □ 2: Palpebral and lower limb edema □ 3: Extreme generalized edema (hyposarca)
Frequency of urination at night	□ 0: None □ 1: 2 times □ 2: 3 to 4 times □ 3: 5 times or more
Constipation	□ 0: None □ 1: Hard stools and straining □ 2: Hard stools with a 2 to 3 days between bowel movements □ 3: Hard stools with 3 or more days between bowel movements
Hematuria	□ 0: None □ 1: Yes

After being screened for inclusion and exclusion criteria (Table 2), eligible patients will be entered into the trial.

**Table 2:** Eligibility criteria

Inclusion criteria	Exclusion criteria
• DKD diagnostic criteria: a) Type 2 diabetes b) Diabetic retinopathy c) 24-h urinary protein (24 h UP) 0.5–3.5 g • Serum creatinine <265 μmol/L (3 mg/dl) • Controlled hypertension (blood pressure ≤ 130/80 mmHg) • A1C ≤ 8.0 • Chinese syndrome differentiation of *qi* and *yin* deficiency with blood stasis • Taking irbesartan (300 mg daily) continuously in the past 2 weeks • Between 18 and 70 years of age • Sign informed consent	• Blood pressure < 90/60 mmHg • Serum potassium > 5.5 mmol/L or < 3.5 mmol/L • Doubling of serum creatinine in the past 6 months • Renal artery stenosis • Severe diabetes complications or serious macrovascular events within 6 months (e.g., cerebral hemorrhage, massive cerebral infarction, or acute myocardial infarction) • Recent infection in the past 4 weeks • Other primary or secondary renal diseases (e.g., IgA nephropathy, membranous nephropathy, or lupus nephritis) • Any malignancy • Severe mental disorder • Pregnant or lactating women or women with the intention of becoming pregnant or women who are not using appropriate contraceptive methods • Allergic to components of agents used in the study • Participation in other clinical trials

### Interventions

Eligible participants will be randomized to either the TSF or placebo group, receiving oral administration of either TSF or placebo (12 g per packet) twice daily for 24 weeks.

TSF consists of seven natural herbs: astragalus root (*Astragalus membranaceus* (Fisch.) Bge.), rehmannia root (*Rehmannia glutinosa* (Gaertn.) Libosch.), notoginseng root (*Panax notoginseng* (Burk.) F. H. Chen), winged burning bush twig (*Euonymus alatus* (Thunb.) Sieb*.*), Asiatic cornelian cherry fruit (*Cornus officinalis* Sieb. Et Zucc.), rhubarb root and rhizome (*Rheum palmatum* L.), and bitter orange (*Citrus aurantium L.*).

Both TSF (Lot No. 1206388) and the placebo were manufactured by the Jiangyin Tianjiang Pharmaceutical Company (Jiangyin, China). The manufacturing process was quality controlled for physical characteristics, purity, microbial content, and weight consistency of the packets. Certificates of quality for the manufacturing of TSF and placebo have been provided by the manufacturer (Additional file 3). The main components in TSF were validated and quantified by thin-layer chromatography and high-performance liquid chromatography.

#### Concomitant treatments

Based on the 2013 American Diabetes Association (ADA) practice guidelines, all participants will receive standard treatments as follows:

1. Irbesartan: Irbesartan will be selected as the basic treatment to lower proteinuria and protect renal function. All participants will take irbesartan orally at a dosage of 300 mg daily.
2. Antihypertensive drugs: Participants with hypertension will receive antihypertensive drugs to maintain a blood pressure of ≤ 130/80 mmHg. Non-dihydropyridine calcium channel blockers or alternatively, beta blockers, will be the recommended drugs. If the blood pressure cannot be maintained at 130/80 mmHg by the combination of these two drugs, diuretics will be added.
3. Blood glucose control therapy: The A1C of all participants will be controlled to ≤ 8.0 % throughout the trial. Metformin will be used for participants with eGFR above 60 ml/min. For participants with eGFR below 60 ml/min, oral repaglinide will be the alternative. For participants whose A1C cannot be controlled with oral agents, insulin will be used.
4. Antilipemic agents: Participants with hyperlipidemia will receive atorvastatin.

Participants will also be instructed to follow a low-sodium diabetes diet, including a reasonable intake of calories, protein, carbohydrates, and fat. They will also be asked to exercise regularly. Administration of other medications will be at the discretion of the attending physician investigator and would not interfere with outcome assessments. Investigators will be instructed to record in the CRF the details of any additional/transformational drug or therapy, such as the name, dosage, and duration of administration.

#### Prohibited concomitant medications

During the trial period, use of any other drugs that could affect urinary protein or renal function will be prohibited. This includes TCMs that function to replenish *qi* and *yin* or promote blood circulation. Other prohibited agents include vitamin D, spironolactone, pentoxifylline, rhubarb sodium bicarbonate tablets, aminoglycoside antibiotics, nonsteroidal anti-inflammatory drugs, and antiviral drugs. If participants use any of the above-mentioned drugs, they will be withdrawn from the study.

### Study visits and assessment

Study duration will be 50 weeks, comprised of a 2-week run-in period, 24 weeks of intervention, and 24 weeks of follow-up. After treatment commences, visits will occur every 4 weeks during the intervention period. During the follow-up period, participants will be asked to visit at 28, 36, and 48 weeks. An overview of specific measurements and time points of data collection can be found in Table 3.

**Table 3:** Schedule of enrollment, allocation, visits, and assessments

	Study Period										
	Enrollment	Allocation	Intervention						Follow-up		
Time point (weeks)	−2	0	4	8	12	16	20	24	28	36	48
Eligibility screen	√										
Written informed consent	√										
Medical history	√										
Physical examination		√	√	√	√	√	√	√			
Routine blood and urine		√		√		√		√			
Electrocardiogram		√		√		√		√			
ALT, AST, ALP, GGT, TBIL		√		√		√		√			
24 h urinary protein		√	√	√		√		√	√	√	√
Serum Cr, eGFR		√	√	√		√		√	√	√	√
TC, TG, LDL-C, HDL-C		√	√	√		√		√			
A1C, CRP		√		√		√		√			
Evaluation of TCM syndrome		√	√	√	√	√	√	√	√	√	√
Drug distribution		√	√	√	√	√	√				
Retrieval of unused drugs			√	√	√	√	√	√			
Adverse events			√	√	√	√	√	√	√	√	√

*Abbreviations*: *A1C* hemoglobin A1C, *ALP* alkaline phosphatase, *ALT* alanine transaminase, *AST* aspartate aminotransferase, *eGFR* estimated glomerular filtration rate, *GGT* gamma-glutamyl transpeptidase, *LDL-C* low-density lipoprotein cholesterol, *serum Cr* serum creatinine, *TBIL* total bilirubin, *TC* total cholesterol, *TCM* traditional Chinese medicine, *TG* triglycerides, *HDL-C* high-density lipoprotein cholesterol

√ = Action items

### Outcome measurements

#### Primary endpoint

The primary endpoint is 24 h UP. The change in 24 h UP from baseline to 24 weeks will be the analysis metric for the difference between the two groups. At baseline and treatment endpoint (24 weeks), the average values of two different samples collected within 1 week will be used to minimize variation between the different urine samples.

#### Secondary endpoints

Secondary endpoints will be as follows:

1. Change in serum creatinine (SCr) and eGFR (2009 CKD-EPI creatinine equation [25]) from baseline to 24 weeks
2. Change in blood lipid profile of total cholesterol (TC), triglyceride (TG), high-density lipoprotein cholesterol (HDL-C), and low-density lipoprotein cholesterol (LDL-C) from baseline to 24 weeks
3. Improvement in TCM symptoms (evaluation by TCM symptom score scale) from baseline to 24 weeks
4. Safety assessment: Results of routine blood and urine tests, electrocardiogram (ECG), alanine aminotransferase (ALT), aspartate aminotransferase (AST), alkaline phosphatase (ALP), gamma-glutamyl transpeptidase (GGT), and serum total bilirubin (TBIL) tests will comprise the comprehensive safety assessment. The safety endpoints will include the incidence and severity of drug-induced liver injury (DILI) and bone marrow suppression (BMS), as well as the incidence of QTc prolongation, as measured by ECG. DILI is defined by criteria established by the International Serious Adverse Events Consortium [26]. The causality relationship between DILI and the intervention drug will be assessed with the Roussel Uclaf Causality Assessment Method (RUCAM) [27]. Severity is scored on a range from 1 (mild) to 5 (fatal) [28]. The diagnosis and severity of bone marrow suppression will be assessed by criteria established by the World Health Organization Collaborating Centre for International Drug Monitoring [29]: grade I: white blood cell (3.0–3.9) × 109/l, hemoglobin 95–100 g/l, platelet (75–99) × 109/l; grade II: white blood cell (2.0–2.9) × 109/l, hemoglobin 80–94 g/l, platelet (50–74) × 109/l; grade III: white blood cell (1.0–1.9) × 109/l, hemoglobin 65–79 g/l, platelet (25–49) × 109/l; grade IV: white blood cell (0–1.0) × 109/l, hemoglobin < 65 g/l, platelet < 25 × 109/l. QTc longer than 0.44 second is regarded as prolongation.

#### Additional measurements

Tests for A1C and serum C-reactive protein (CRP) will be carried out at study visits 4, 8, 16, and 24 weeks.

#### Adverse events

Adverse events (AEs) such as signs and symptoms and other ailments will be documented at every study visit. Each AE will be classified as a mild, moderate, or severe AE, and its correlation with the intervention drugs will be assessed. Severe AEs will be reported to the principal investigator and the ethics committee within 24 h. All the adverse events will be recorded, monitored, and treated until properly resolved.

### Quality control of laboratory specimens

To eliminate potential discrepancies resulting from the handling of laboratory specimens by different labs, which may compromise data quality, samples collected at patient visits from all five participating medical centers will be cold-chain transported to a single laboratory (KingMed Diagnostics, Guangzhou) for processing. Data from KingMed will be quality controlled and securely transmitted in batches via e-mail back to participating sites and the principal investigators.

Blood and urine samples will be collected on site by a trained phlebotomist using standard techniques. The serum of each blood sample will be separated by centrifugation (3000 g, 15 minutes, 4 °C). Samples will be analyzed by an automated biochemistry analyzer (Roche Modular P800, Roche Diagnostics, Lewes, UK) using validated commercial kits. The 24 h UP and CRP will be assayed by immunoturbidimetry. Serum levels of sodium, potassium, and chlorine will be detected by ion-selective electrode methodology. Bilirubin will be determined by the diazo method. ALT, AST, ALP, and GGT will be measured by the velocity method. A direct method will be used for the quantitative determination of LDL-C and HDL-C. An enzymatic method will be used to assay SCr and TG. Cholesterol will be measured by the cholesterol oxidase method.

### Sample size estimation

The sample size was estimated based on results from our preliminary trial. These results found that after a 24-week treatment period, the mean reduction in 24 h UP in the placebo group was 0.40 ± 0.91 g; the mean reduction in 24 h UP in the TSF group was −0.31 ± 0.95 g. The least-squares mean of the difference value between the relative changes from baseline (TSF group-placebo group) was −0.54. The proportion of the cases between the TSF group and placebo group will be 1:1. With 80 % power to detect and a two-sided level of significance of 0.05, the sample size was estimated to be 54 in each group. In order to increase the accuracy of the data obtained, this number was increased to 60. Assuming a dropout rate of 20 %, the final sample size was estimated to be 144.

### Randomization and concealment

Randomization will be carried out by an independent clinical research organization (CRO) from the Beijing Qihuang Drug Clinical Research Center. The CRO will use a computerized random number generator to produce the randomization schedule employing permuted blocks of random sizes. Participants will be randomly assigned to either the control or the TSF group at a 1:1 allocation. The drug administrator at each participating medical center will enroll patients sequentially based on consultation order. The blocks should provide comparable numbers of participants in both groups at any time in the course of the trial. To ensure concealment, the block sizes will not be disclosed. The randomization list will be retained by the Beijing Qihuang Drug Clinical Research Center for the duration of the study.

### Blinding

This study will implement a double-blind design in which participants and investigators will be blinded to treatment allocation. The statistician will not be involved in randomization and will analyze data without having access to information about allocation. Treatment allocation will be disclosed after the conclusion of the trial except in cases of emergency, such as severe AEs. Both TSF and placebo are manufactured as fine brown granules and packaged in packets identical in appearance. When dissolved in warm water, the taste, smell, and appearance of the TSF and placebo are identical. Investigators and participants will be unaware of the drug allocation.

### Statistical analysis

This trial will use three analysis sets: the intention-to-treat set, which will regard all participants as randomized regardless of whether or not they received the randomized treatment; the per protocol analysis set, which will be restricted to participants who complete the 24-week treatment and/or have the primary endpoint measurement records of at least three time points during the study; and the safety analysis set, which will include randomized participants who have made at least one visit. Multiple imputation analysis will be performed to account for the effect of missing data. Partially missing data will be carried forward with the principle of the last observation carried forward (LOCF). A *P* value less than 0.05 will be considered significant. SAS 9.2 software (SAS Institute, Cary, NC, USA) will be used for analysis.

Continuous variables will be expressed as the mean (SD), and categorical variables, as numbers and percentages. To compare baseline data between the two groups, independent *t* tests (or the Mann–Whitney U test) will be used to analyze continuous data, and chi-square or Fisher exact test will be used for categorical data. Outcome analysis will be performed using *t* test for normally distributed data and rank sum test for non-normal distribution data. Considering that the baseline 24 h UP and/or A1C are essential for the progression of urinary protein, analysis of covariance (ANCOVA) using 24 h UP and/or A1C as covariates will be performed to analyze the 24 h UP reduction at 24 weeks. To investigate the effects of treatment and time course, repeated measures analysis of variance (ANOVA) will be applied to determine changes in the 24 h UP and eGFR at each visit. For safety endpoints, incidences of DILI and BMS will be analyzed by Kruskal-Wallis test, and the incidence of QTc will be evaluated by a chi-square test. For AE evaluation, all AEs will be reported including its grade, time of occurrence, duration, treatment, prognosis, and correlation with the intervention drugs. The chi-square test or Fisher exact test will be used to compare the incidence of AEs between the two groups.

### Data collection and management

Investigators at all sites will be trained uniformly in standard operating procedures (SOPs) for trial execution, interrogation of TCM symptoms, laboratory specimen collection, and handling. The CRO will pay regular visits to each site to confirm protocol adherence and the quality of data collection, and resolve any issues the site has. To ensure the accuracy of data entry, non-numeric data will be coded for ease of data storage; analysis and referential data rules, valid values, range checks, and consistency checks against data already stored in the database will also be supported. After verification of the content of the written CRFs, data will be input into the database independently by two full-time research staff. All documents will be stored safely under confidential conditions and archived for 5 years.

### Participant retention and withdrawal

To prevent missing data and to avoid associated complexities in the study analysis, once a patient is randomized, investigators will make reasonable efforts to follow the patient for the entire study period. Efforts will include the following:

1. Reimbursement of participants for public transportation costs
2. Scheduling and reminding participants of their return visits
3. Reimbursing participants in the placebo group at the conclusion of the trial

Participants may withdraw from the study for any reason at any time, and the reason will be recorded in the CRF. Investigators can also withdraw participants from the study to protect their safety. Reasons that can prompt withdrawal include the following:

1. Rapidly increasing serum creatinine of more than 50 % of baseline after 2 to 4 weeks of treatment
2. Decreasing eGFR of more than 50 % of the baseline after 2 to 4 weeks of treatment
3. Development of severe complications and/or deterioration of an existing health condition

## Discussion

TCM is a millennia-old medical practice based on syndrome pattern differentiation and is applied to treat a variety of diseases in China. In modern times, TCM is prescribed by most doctors in China as a basic or complementary therapy for kidney disease. TSF is prescribed to treat DKD patients with the most common syndrome pattern of deficiency in *qi* and *yin*. Laboratory studies and clinical observations have shown that TSF appears to be efficacious [17, 18]. We are therefore undertaking this trial to validate its efficacy and safety for the treatment of DKD.

The quality of the RCTs conducted on TCM therapies is generally poor [30]. Issues include poor study design and methodology, and a lack of training of the investigators and staff. To ensure the quality of this study and reliability of our conclusions, the experimental design and study implementation have been carried out to date under strict quality control. Statisticians are involved in the study design to conduct sample size estimation, random allocation sequence generation, randomization concealment, and blinding. The Consolidated Standards of Reporting Trials (CONSORT) Extension for Herbal Interventions [31] has been used to monitor other points of the study design. A training session for each center has been held to explain the study protocol, explain how the TCM syndrome pattern differentiation should be performed, and clarify the SOPs. An independent laboratory will process the biochemical measurements. Additionally, the trial progress and quality will be monitored by a trained CRO.

The findings of this study may provide an alternative treatment option for DKD patients with macroalbuminuria. It will also provide evidence and a scientific rationale for the use of TSF to treat the macroalbuminuria stage of DKD.

## Trial status

The trial is currently ongoing and 123 patients have been recruited.

## Additional files

Additional file 1: SPIRIT checklist. (DOC 121 kb) Additional file 2: Consent form. (PDF 361 kb) Additional file 3: Certificates of manufacturing quality of TSF and placebo. (PDF 270 kb)

## Abbreviations

CRP: C-reactive protein
DILI: drug-induced liver injury
DM: diabetes mellitus
ECG: electrocardiogram
eGFR: estimated glomerular filtration rate
ESRD: end-stage renal disease
GGT: gamma-glutamyl transpeptidase
HDL-C: high density lipoprotein-cholesterol
LDL-C: low-density lipoprotein cholesterol
LOCF: last observation carried forward
MMP-9: matrix metallopeptidase 9
NKF K-DOQI: National Kidney Foundation Kidney Disease Outcomes Quality Initiative
OLETF: Otsuka Long-Evans Tokushima fatty
RCT: randomized controlled trial
RUCAM: Roussel Uclaf Causality Assessment Method
SCr: serum creatinine
SOPs: standard operating procedures
TBIL: serum total bilirubin
TC: total cholesterol
TCM: traditional Chinese medicine
TG: triglyceride
TGF-β1: transforming growth factor beta 1
UAER: urinary albumin excretion rate
