# Supplementary material for: Chinese herbal medicine Tangshen Formula treatment of patients with type 2 diabetic kidney disease with macroalbuminuria: study protocol for a randomized controlled trial
Source: Trials. 2016 May 23;17:259. doi: 10.1186/s13063-016-1385-2 (PMC4877744; doi:10.1186/s13063-016-1385-2)
Supplement: Additional file 2: — Consent form. (PDF 361 kb) [file 13063_2016_1385_MOESM2_ESM.pdf]

糖肾方配方颗粒临床研究知情同意书

Consent Form of Tangshen Formula Clinical Trial

尊敬的受试者:

Dear participants:

为了提高糖尿病肾病的防治水平，避免严重并发症的发生，我们在“十二五”期间，获得国家科技部支撑计划课题和国家科技合作等项目的资助（项目编号：2013BA102B04；2011DFA31860；81130066）。因为您具备①确诊为糖尿病肾脏疾病；②24h 尿蛋白：0.5～3.5g；③血清肌酐＜265μmol/L（3mg/dl）；④中医辨证符合气阴两虚夹瘀；⑤血压≤130/80mmHg、HbA1c≤7.5% 的研究入组条件。我们邀请您参加**《糖肾方治疗糖尿病肾病有效性和安全性的随机、双盲、安慰剂平行对照、多中心临床试验研究》**，本研究已经得到中日友好医院伦理委员会的审查和批准。

请仔细阅读本知情同意书并慎重做出是否参加研究的决定。我们鼓励您在做出参与此项研究的决定之前，和您的家人及朋友进行充分讨论。

1. 为什么进行这项研究？

What is the purpose of this study?

研究背景(background)：糖尿病肾病有几个重要的阶段：微量白蛋白尿(尿白蛋白排泄率为 20-200μg/min 或 30-300mg/24h)，大量蛋白尿期（大于 300mg/d 或大于 200μg/min），终末期肾病。患者一旦进入大量蛋白尿期出现蛋白尿，则提示出现肾功能减退将不可避免。目前国际上尚缺少延缓糖尿病肾病病程进展的特效药物，西药血管紧张素Ⅱ受体拮抗剂被证实有效而受到关注。但是本课题组前期在国家基础研究计划 973 中医基础理论专项课题和首都医学发展科研基金管理委员会的资助下，发现中药糖肾方配方颗粒在使用西药常规治疗的基础上能进一步降低 24h 尿蛋白定量，并提高肾小球滤过率。

研究目的(objectives)：科学评价糖肾方治疗糖尿病肾病临床蛋白尿期的临床疗效和安全性，并探索糖肾方治疗糖尿病肾病大量蛋白尿的作用机理。

2. 多少人将参与这项研究？

How many patients will participate in this study?

大约 144 人将参与在中日友好医院、天津中医药大学第一附属医院、上海龙华医院、杭州市中医院、陕西省中医院等 5 家三级甲等医院开展的本项研究。

3. 本研究包括哪些内容？

What are the main contents of this study?

如果您同意参加本研究，您将被随机分配到不同治疗方案两组中的任意一组，一组是西药常规治疗+糖肾方配方颗粒，一组是西药常规治疗+糖肾方模拟剂。其中西药常规治疗措施，包括医生对您饮食的指导、降糖、降压及调节血脂等糖尿病肾病常规使用的治疗方法。您有 50% 的几率使用本研究药物。

本研究将持续给药观察 24 周共 6 次访视，在此期间，您需要每 4 周来医院做一些相关检查，告诉我们您的任何变化，并给您发放随后 4 周的药物。治疗周期结束后，我们将对您进行为期 24 周的随访观察，观察内容主要包括 24 小时尿蛋白定量及血肌酐的免费检测及相关的治疗指导。

疗效性评价指标

- 24 小时尿蛋白定量：筛选期、基线、给药 4、8、16、24 周各检查 1 次。（**在基线和 24 周时分别 1 周内检测 2 次，取平均值。**）
- 肾功能（Scr/ CKD-EPI 公式计算 eGFR）：筛选期、基线、给药 4、8、16、24 周各检查 1 次；
- 中医证候积分：基线、给药 4、8、12、16、20、24 周各记录 1 次；
- 血脂(TG、TC、LDL、HDL)：筛选期、基线、给药 8、16、24 周各检查 1 次；
- 炎性指标（hs-CRP、MMP-1/MMP-9）、氧化应激指标（8-oxodG）：在基线、给药 8、16、24 周各留 1 次血、尿样本，待试验结束后统一检测。

安全性评价指标

- 生命体征（血压、呼吸、心率、心律、体温）：筛选期、基线、给药 4、8、12、16、20、24 周各检查、记录 1 次；
- 血、尿常规；电解质（K、Na、Cl）；肝功能（ALT、AST、GGT、ALP、TBIL）；心电图：基线、给药 8、16、24 周各检查 1 次；
- 不良事件：随时详细记录。

4. 研究中我该做什么？

What would I have to do?

研究期间，①.您需要按日程进行回访，并告诉我们您的任何变化。②.严格按医师医嘱的要求，按时、按量服用药物；③.观察期间禁止服用其他治疗糖尿病肾病的中、西药物；④.合并其他疾病所必须继续服用的药物可维持不变，但应在主管医师的指导下应用；在服用任何新的处方药物前请咨询您的研究医生；⑤.育龄期妇女应同意在参加本项研究期间采取有效避孕措施；⑥.在整个研究期间您不能再参加其他任何有关药物或者医疗器械的临床研究。

5. 参加本研究的风险是什么？

What are the risks?

参加本研究可能给您带来的风险如下。您应该和您的研究医生，或者您愿意，与您平日照看您的医生讨论一下这些风险。

药物不良反应(drug induced adverse events)：虽然我们前期研究中糖肾方未发现药物不良反应，但由于对于药物的个体反应不同，受试者在药物观察研究过程中，可能会出现各种不适情况；参与临床观察期间，您需要和研究医生保持联系，并且坚持服用药物，完成必要的生理生化检查。

抽血的风险（risk of blood collection）：从胳膊抽血的风险包括短暂的不适和/或青紫。

药物相互作用（drug interactions）：安全起见，您必须在研究开始前告诉研究医生您正在服用的所有处方药、中药制品、非处方药、维生素和天然补品等保健品。在研究过程中需要服用这些药物之前也请务必告诉您的研究医生。

6. 参加研究有什么受益？

What benefit will I receive?

如果您同意参加本研究，您的糖尿病肾病可能会及到有效的治疗，有利于延缓疾病进展。

从本研究取得的研究成果将有益于糖尿病肾病的治疗，惠及全国更多的同类患者。

7. 我的信息会保密吗？

Will my personal information be kept confidential?

我们会按照法律的要求为您的研究记录保密。我国的相关法律为隐私、数据和授权访问的安全提供了保障。您的医疗记录将保存在医院，研究者、研究主管部门、伦理委员会将被允许查阅您的医疗记录。任何有关本项研究结果的公开报告将不会披露您的个人身份。

8. 如果我在参加研究期间受到损害怎么办？

What if I get hurt during participation in this study?

如果您的健康确因参加这项研究而发生与研究相关的损害，请立即通知研究医生，他们将负责对您采取适当的治疗措施。在专门机构鉴定认可后，申办者将承担治疗费用及按国家有关规定对您给予相应的经济补偿。对因医疗事故或因未遵循研究方案程序而导致的损伤，申办者不予补偿。

即使您已经签署这份知情同意书，您仍然保留您所有的合法权利。

9. 我能得到什么补偿？

Will I be paid by participation?

您不会因参加本研究而获得任何酬劳。为了补偿您参加本研究可能给您带来的不便，您将得到一定的交通补贴，除此之外，在研究期间，您可免费得到研究药物并进行试验期间部分指标的检查。

10. 拒绝参加或者退出研究的权利

The right of declining to participate or withdraw

参加本研究是完全自愿的，您可以拒绝参加研究，或者研究过程中的任何时候选择退出研究，不需任何理由。该决定不会影响您未来的治疗。

如果您决定退出本研究，请提前通知您的研究医生。为了保障您的安全，您可能被要求进行相关检查，这对保护您的健康是有利的。

除此之外，您必须归还所有未用的研究药品给研究医生。

如果您的状况更差了，或者您出现严重的不良反应，或者您的研究医生觉得继续参加研究不符合您的最佳利益，他/她会决定让您退出研究。

11. 如果我有问题或困难，该与谁联系？

Who can I contact, if I have further questions?

如果您有与本研究相关的任何问题，请联系李平教授或严美花医生，工作时间请拨打 010-84205633,下班时间、周末和节假日拨打 15810063103。

如果您有与自身权利/权益相关的任何问题，或者您想反映参与本研究过程中遭遇的困难、不满和忧虑，或者想提供与本研究有关的意见和建议，请联系中日友好医院伦理审查委员会，联系电话：010-84205708。

告知声明

Statement of information

“我已告知该受试者《糖肾方治疗糖尿病肾病有效性和安全性的随机双盲、安慰剂平行对照、多中心临床试验研究》的研究背景、目的、步骤、风险及获益情况，给予他/她足够的时间阅读知情同意书、与他人讨论，并解答了其有关研究的问题；我已告知该受试者当遇到与研究相关的问题时可随时与李平教授或严美花医生联系，遇到与自身权利/权益相关问题时随时与中日友好医院医学伦理委员会联系，并提供了准确的联系方式；我已告知该受试者他/她可以退出本研究；我已告知该受试者他/她将得到这份知情同意书的副本，上面包含我和他/她的签名。”

研究者签名（signature of investigator）：__________ 日期(date)：__________

知情同意声明

Informed consent statement

“我已被告知《糖肾方治疗糖尿病肾病有效性和安全性的随机双盲、安慰剂平行对照、多中心临床试验研究》的研究的背景、目的、步骤、风险及获益情况。我有足够的时间和机会进行提问，问题的答复我很满意。我也被告知，当我有问题、想反映困难、顾虑、对研究的建议，

或想进一步获得信息，或为研究提供帮助时，应当与谁联系。我已经阅读这份知情同意书，并且同意参加本研究。我知道我可以在研究期间的任何时候无需任何理由退出本研究。我被告知我将得到这份知情同意书的副本，上面包含我和研究者的签名。”

受试者签名（signature of participant）：____________ 日期(date)：____________

受试者联系电话(phone number)：______________
