# Supplementary material for: Chinese herbal medicine Tangshen Formula treatment of patients with type 2 diabetic kidney disease with macroalbuminuria: study protocol for a randomized controlled trial
Source: Trials. 2016 May 23;17:259. doi: 10.1186/s13063-016-1385-2 (PMC4877744; doi:10.1186/s13063-016-1385-2)
Supplement: Additional file 3: — Certificates of manufacturing quality of TSF and placebo. (PDF 270 kb) [file 13063_2016_1385_MOESM3_ESM.pdf]

[redacted]

江阴天江药业有限公司检验报告单

[redacted]

NO. 18894

代号	0	品名	[redacted] 糖肾方颗粒(药) 配方颗粒
[redacted] 批号	1206388	规格	12克/袋
依据	江阴天江药业有限公司暂行质量标准	数量	22320袋
取样日期	2012-7-23	报告日期	2012-10-24

检查项目	标　准	结　果
[性　状] [redacted]	本品为棕色颗粒，气微，味微苦。	
[水　分] [redacted]	≤6.0%	3.1%
[溶化性] [redacted]	溶化，不得有焦屑，允许有轻微浑浊	符合规定
[装量差异] [redacted]	装量差异限度　±5%	符合规定
[微生物限度检查] [redacted]		
细菌数:	<1000cfu/g	10cfu/g
霉菌和酵母菌数:	<100cfu/g	<10cfu/g
大肠埃希菌:	不得检出/g	未检出

江阴天江药业有限公司 检验专用章

结　论	本品按江阴天江药业有限公司暂行质量标准检验，结果符合规定

负责人 朱文印　复核人 仁江单平　检验人 彦芳王　梅王印亚

江阴天江药业有限公司检验报告单

NO. 18876

代号	0	品名	[redacted] 糖肾方颗粒(安) 配方颗粒
批号	1206388.	规格	12克/袋
依据	江阴天江药业有限公司暂行质量标准	数量	22920袋
取样日期	2012-7-21	报告日期	2012-10-24

检查项目	标　　准	结　　果
[性　状]	本品为棕色颗粒,气微,味微苦。	
[水　分]	≤6.0%	3.1%
[溶化性]	溶化,不得有焦屑,允许有轻微浑浊	符合规定
[装量差异]	装量差异限度　±5%	符合规定
[微生物限度检查]		
细菌数:	<1000cfu/g	50cfu/g
霉菌和酵母菌数:	<100cfu/g	<10cfu/g
大肠埃希菌:	不得检出/g	未检出

江阴天江药业有限公司 检验专用章

结　论	本品按江阴天江药业有限公司暂行质量标准检验,结果符合规定

负责人　　　　复核人　　　　检验人
